# Chemical signal is in the blend: bases of plant-pollinator encounter in a highly specialized interaction

**DOI:** 10.1038/s41598-020-66655-w

**Published:** 2020-06-22

**Authors:** Magali Proffit, Benoit Lapeyre, Bruno Buatois, Xiaoxia Deng, Pierre Arnal, Flora Gouzerh, David Carrasco, Martine Hossaert-McKey

**Affiliations:** 1 CEFE, Univ Montpellier, CNRS, EPHE, IRD, Univ Paul Valéry Montpellier 3, Montpellier, France; 2 MIVEGEC, Univ Montpellier, IRD, CNRS, Montpellier, France

**Keywords:** Behavioural ecology, Chemical ecology, Analytical chemistry

## Abstract

In several highly specialized plant-insect interactions, scent-mediated specificity of pollinator attraction is directed by the emission and detection of volatile organic compounds (VOCs). Although some plants engaged in such interactions emit singular compounds, others emit mixtures of VOCs commonly emitted by plants. We investigated the chemical ecological bases of host plant recognition in the nursery pollination mutualism between the dioecious *Ficus carica* and its specific pollinator *Blastophaga psenes*. Using Y-tube olfactometer tests, we show that *B. psenes* females are attracted by VOCs of receptive figs of both sexes and do not exhibit preference for VOCs of either male or female figs. Electrophysiological tests and chemical analysis revealed that of all the VOCs emitted by receptive figs, only five were found to be active on female antennae. Behavioural tests show that, in contrast to VOCs presented alone, only a blend with a particular proportion of four of these VOCs is as attractive as the odour of receptive figs, and that if there is a very small change in this blend proportion, the pollinator is no longer attracted. This study revealed that in highly specialized mutualistic interactions specificity could be mediated by a particular blend of common compounds emitted by plants.

## Introduction

About two‐thirds of all flowering plants depend on insects for pollination^1^. Plant-pollinator encounters are mediated by the different cues plants display, generally visual and olfactory, that constitute signals for their pollinators. For instance, floral volatile organic compounds (VOCs) are generally involved in the attraction of pollinators independently of the degree of interaction specialization^2,3^. Within the complex VOCs mixtures emitted by plants, pollinators only detect a part of the compounds and use a portion of them as a signal to find their resource^2,4,5^. In specialized plant-insect interactions, partner encounter should be mediated by particular floral signals that allow pollinators to unambiguously identify their host-plants. Therefore, it has been hypothesized that scent-mediated specificity of pollinator attraction to plants is directed by the emission and detection of either i) uncommon compounds emitted by plants or ii) a blend of common compounds emitted in unique proportions^2,4,6^. For instance, the interaction between diverse groups of floral oil-secreting plants and oil-collecting bees around the world is mediated by one rare VOC, diacetin^7^. Unique compounds have also been documented as pollinator attractants in sexually deceptive orchids, for example in several orchid species of the genus *Chiloglottis*^8,9^. However, other sexually deceptive orchid species emit blends of commonly occurring hydrocarbons to attract their specific pollinators^10^. While the nature of the chemical signals responsible for the specific attraction of pollinators has been well studied in the case of sexually deceptive Australian and European orchids^9–14^, there are still major gaps in our understanding of the signals involved in plant-pollinator encounter in most specialized interactions.

Nursery pollination mutualisms, in which larvae of the pollinators feed on floral tissue, are among the most specialized plant-pollinator interactions. In several of these interactions, empirical studies have pointed out the determinant role of floral VOCs for the attraction of the highly specialized and obligate pollinators^3,6,15–17^. Behavioural studies conducted in the laboratory have shown for three different nursery pollination systems that pollinators are significantly attracted by one or two major VOCs emitted by their respective host plants, which are uncommon compounds^6,16,17^. However, chemical analysis of floral VOCs indicates that not all plants involved in nursery pollination mutualisms emit rare compounds^3^. This therefore suggests that the specific attraction of the pollinator in these interactions is mediated by common plant-emitted VOCs.

In the 800 interactions between *Ficus* species (Moraceae) and their pollinating fig wasps (Hymenoptera, Chalcidoidea, Agaonidae) pollinators reproduce within the flowers of the inflorescence, *i.e*. the fig, they pollinate. Inflorescences of *Ficus* species emit complex species-specific mixtures of VOCs that attract specifically their pollinators^3,6,18–25^. However, except for one species^6^, the composition of the chemical signal responsible for the specific attraction of pollinating fig wasps to figs of their host species is still unidentified.

Pollinators of all dioecious *Ficus* species (roughly 50% of all *Ficus* species) suffer a conflict of interest with their host plant because they cannot reproduce within female figs, which are therefore pollinated by deceit^3,26^. From a theoretical point of view, selection should favour those wasps that are able to distinguish between female and male tree figs, so that they enter solely into the latter. However, if wasps avoid female tree figs (*i.e*. no pollination), this would lead to the end of fig seed production and to a potential breakdown of the dioecious system in figs.

Different hypotheses have been proposed to explain the persistence of the mutualism in dioecious fig species and widely discussed in other papers^3,26^. One of the hypotheses proposes that pollinating fig wasps cannot choose between male and female tree figs because they are not able to differentiate between them^26,27^. Indeed, a recent study describing the VOCs emitted by several dioecious *Ficus* species revealed an apparent intersexual similarity in those species in which male and female figs are receptive to pollinators at the same time^28^. This could explain why pollinators seem unable to discriminate between male and female receptive figs^26,27^. However, appropriate behavioural bioassays to test this hypothesis are still lacking.

**Figure 1:**
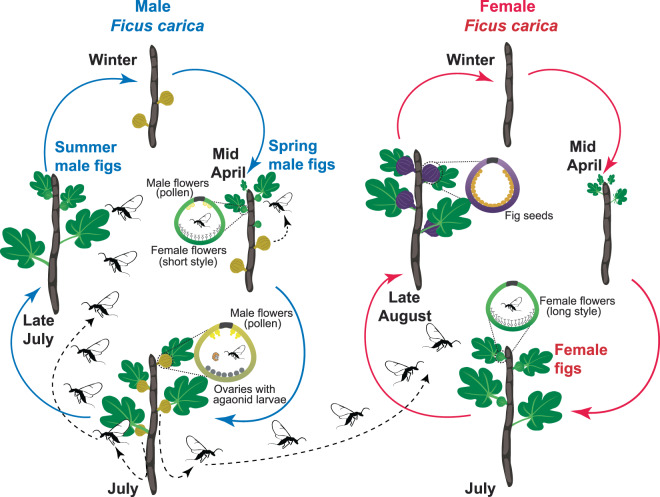
Life cycles of male and female tree of *Ficus carica* (respectively on the left and right side) and *Blastophaga psenes* in southern France. For each type of fig, receptive (green), ripe male (khaki) and ripe female (purple), a schematic representing wasps and flowers inside the fig is presented. Maturing male figs give rise to wasps (grey) and pollen (yellow), whereas female figs produce only seeds (orange) and contain no male flowers. Females and males of *B. psenes* are also represented in black and brown respectively. *Blastophaga psenes* has two generations per year coinciding with the flowering of male trees first in April (spring male figs) and then in July (summer male figs). In contrast, female trees flower only once a year, in July, and thus partially synchronously with summer male figs. The two distinct productions of male figs perform different functions. *Blastophaga psenes* larvae survive winter by staying in diapause within summer-produced male figs that will stay on the tree until the following spring. In spring, the overwintering wasps complete their development and male pollinators emerge in the fig cavity and copulate with female wasps before the latter emerge from their galls. After emerging from the gall within the fig cavity, female wasps exit their natal figs to enter the spring male figs, in which they oviposit. In summer, the new generation of adult female wasps, after having been fertilized, exit from their natal figs loaded with pollen grains. At this point in time, figs of male and female trees have reached receptivity, and female wasps face two scenarios: i) penetrate into figs of female trees, pollinate their flowers and then die without laying eggs due to a morphological incompatibility between the wasp ovipositor and the style of the female flowers, or ii) penetrate into figs of male trees and reproduce by laying eggs within the ovaries. Then, the cycle closes when the female offspring that entered male figs exit from them later in summer and find male figs in which they will oviposit, giving rise to the overwintering generation. This figure was prepared with the help of Jennifer McKey.

In the present study, we investigate the basis of plant-pollinator chemical communication in the specific interaction between the pollinating wasp, *Blastophaga psenes*, and the dioecious Mediterranean fig tree, *Ficus carica*. Volatile odour profiles of male and female receptive figs of *F. carica* have already been described^29,30^. As in most *Ficus* species, these profiles are constituted by numerous compounds (26, in the case of *F. carica*) commonly occurring in floral scents^29–31^. Based on these results, we hypothesized that receptive figs of *F. carica* emit a blend of common VOCs in a particular proportion to attract *B. psenes*. *Ficus carica* is a dioecious species that displays an unusual phenology: in summer, both sexes flower partly synchronously, whereas in spring only male trees flower (see Fig. 1). Previous chemical analyses conducted on the odour of receptive figs reported that chemical profiles of summer male figs resemble those of the co-flowering females, and are different from those of spring male figs, when female figs are absent. Based on these results we hypothesized that the pollinator-attractive blend of VOCs is similar between male and female figs in summer, so that pollinators cannot discriminate between these two types of inflorescences. In order to test our hypotheses, we combined chemical and electrophysiological analysis with behavioural tests using both natural and synthetic VOCs. We addressed the following questions: (1) Does *B. psenes* discriminate VOCs emitted by male and female receptive figs of *F. carica*? (2) Does *B. psenes* use a combination of common VOCs to find its host plant? (3) Is the proportion of the different VOCs emitted important for host plant recognition?

## Results

### Does the pollinator discriminate between odours of male and female receptive figs?

**Figure 2:**
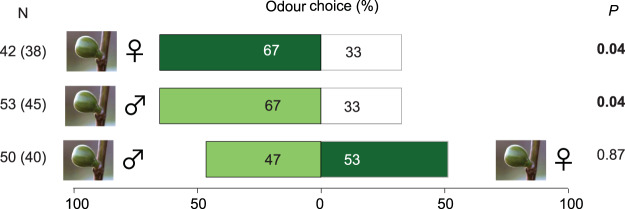
Attraction responses of *Blastophaga psenes* females towards VOCs of summer-male and female receptive figs. Tests were conducted in Y-tube olfactometers where females were allowed to choose between VOCs of either female or male receptive figs in one branch, and control odour in the other. In a second set of tests, female wasps could choose between odours of female and male receptive figs. Number of wasps tested (N), number of individuals that made a choice (in parentheses), and *P*-values (exact binomial test) are indicated for each comparison. Photograph by D. Delgado.

In our olfactometer bioassays, females of *B. psenes* were significantly more attracted by VOCs released by both summer male (binomial test, N = 38, *P* = 0.04) and female receptive figs (binomial test, N = 45, *P* = 0.04), than by the control (empty container) (Fig. 2). In addition, pollinators did not show any significant preference for either summer male or female figs when these were presented at the same time on different arms of the Y-tube olfactometer (Fig. 2, binomial test, N = 40, *P* = 0.87).

### Among the VOCs emitted by receptive figs, which compounds are active on *B. psenes* antennae?

**Figure 3:**
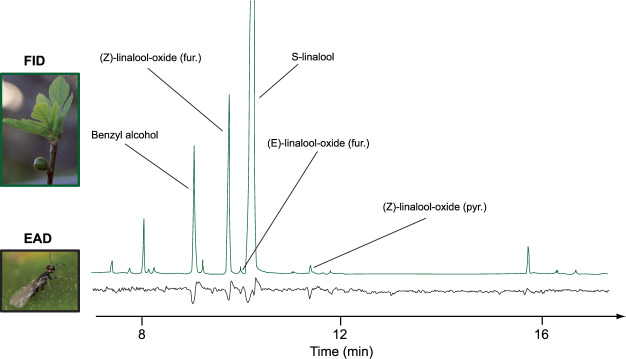
Example of response of antennae of *Blastophaga psenes* females to VOCs from receptive figs. Analyses were carried out using gas chromatography coupled with electroantennographic detection (GC-EAD). Bottom, antennal signal (EAD); top, GC trace (FID). Photographs by D. Delgado and F. Kjellberg.

In order to identify which compounds are detected by the pollinator antennae in the complex mixture of VOCs emitted by receptive figs^29,30^, we performed experiments of electroantennographic detection coupled with gas chromatography (GC-EAD). These analyses conducted on *B. psenes* revealed that antennae of the pollinator responded consistently to five compounds present in the headspace of receptive figs of *F. carica*: one shikimic compound, benzyl alcohol; and four monoterpenes, (*S*)-linalool, (*Z*)-linalool-oxide (furanoid), (*E*)-linalool-oxide (furanoid) and (*Z*)-linalool-oxide (pyranoid) (Fig. 3). Reponses to these five VOCs were confirmed with synthetic compounds. Except for (*Z*)-linalool-oxide (pyranoid), these compounds were always present in the volatile profiles of male and female receptive figs. In contrast, (*Z*)-linalool-oxide (pyranoid) was present in fewer than 30% of our samples: in two out of six samples for spring male figs, and only in one out of four samples for male and female summer figs. If a VOC is involved in pollinator attraction it should always be present in the odour emitted by receptive figs. Therefore, (*Z*)-linalool-oxide (pyranoid) was discarded from the rest of our analyses. Proportions of the four other antennal-active VOCs in the different types of figs, as well as the total quantities emitted by receptive figs, are presented in Table 1.

**Table 1:** Composition of the odour sources used for the behavioural test.

	Odour Source	Benzyl alcohol^a^	(*S*)-linalool^b^	(*Z*)-linalool^c^ oxide (fur.)	(*E*)-linalool^c^ oxide (fur.)	Emission rate^d^ (ng.min^−1^)
Figs	Spring male	7.58 ± 0.73	90.22 ± 0.64	2.14 ± 0.30	0.06 ± 0.001	115.84 ± 20.50
	Summer male	27.02 ± 15.31	70.45 ± 14.52	2.44 ± 1.34	0.08 ± 0.05	29.84 ± 17.14
	Female	33.17 ± 9.30	64.26 ± 9.75	2.49 ± 1.77	0.09 ± 0.06	7.41 ± 2.85
	Mean	20.45 ± 5.59	77.15 ± 5.53	2.33 ± 0.59	0.07 ± 0.02	60.32 ± 16.94
Synthetic compounds	Li	0	100	0	0	34.90 ± 4.00
	BA	100	0	0	0	34.43 ± 4.00
	Li Ox	0	0	50	50	20.57 ± 4.11
	B1	22.90	76.34	0.38	0.38	65.92 ± 7.98
	B2	10.53	87.72	0.88	0.88	35.80 ± 5.74
	B3	0	99.01	0.50	0.50	64.35 ± 8.73
	B4	1.94	97.09	0.49	0.49	59.08 ± 10.08
	B5	16.73	83.17	0.05	0.05	126.07 ± 6.62
	B6	18.25	78.85	1.50	1.50	119.55 ± 11.21
	B7	14.82	79.17	3.00	3.00	105.55 ± 8.66

Percentages of the different volatile organic compounds (VOCs) in each odour source are presented, as well as the diffusion rate of the odour source. For the odours of receptive figs, for each odour the means are based on the measurements conducted using headspace sampling and GC-MS analyses. In addition, we present the average (±standard errors) of the three odour sources. For the synthetic compounds, the percentage indicated represents the quantity introduced in the vial and the diffusion rates are based on the measures of the weight-loss of the vial. The synthetic mixtures are as follows: Li (linalool alone), BA (benzyl alcohol alone), Li Ox [(Z) and (E)-linalool oxide furanoid] and B1-B7 (seven blends with mixtures of the four VOCs in different proportions).

^a^Benzyl alcohol (Fluka, CAS Number: 100-51-6; purity: >99.5).

^2^(S)-linalool and (R)-linalool racemic (Fluka, Racemic mixture CAS Number: 78-70-6; purity: ~97%).

^c^Li Ox: (Z) and (E)-linalool oxide furanoid (Fluka, CAS Number: 68780-91-6; purity: >97%).

^d^For the figs, this value corresponds to emission rate per fig.

### Is the emission of antennal-active VOCs different among the three types of figs?

**Figure 4:**
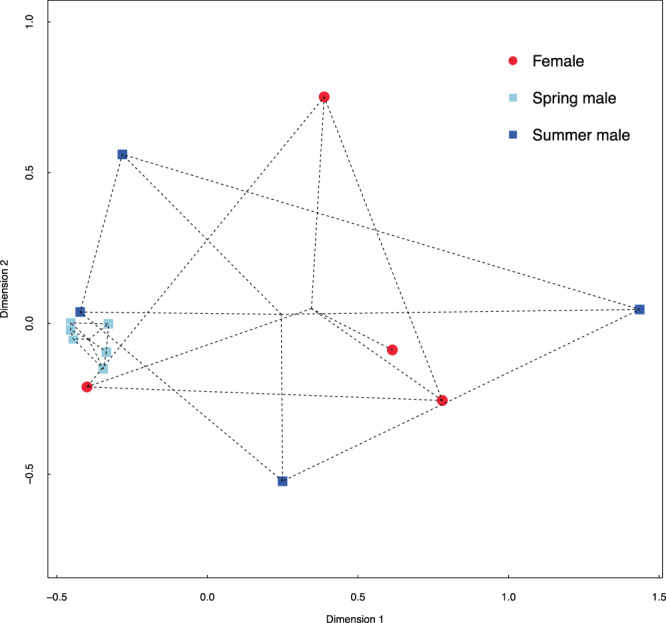
Non-metric multi-dimensional scaling of the relative proportions of VOCs in the global bouquets emitted by the three types of receptive figs of *Ficus carica* based on Bray-Curtis dissimilarity index (stress = 0.03). Male trees flower twice a year, in spring (spring male figs ) and in summer (summer male figs ), and females only during summer (females ). Samples are grouped (dashed lines) by type of fig, and the centroid of each group is indicated.

Emissions of the three types of figs (*i.e*. female, summer male and spring male figs) showed no significant variation in the relative proportions of the four antennal-active compounds (NMDS, Fig. 4, stress = 0.03; PERMANOVA, F_2,13_ = 0.25, *P* = 0.12). Pairwise comparisons pointed out homogeneity between the odour of spring males and summer males (*P* = 0.19), between spring males and females (*P* = 0.10) and between female and summer male figs (*P* = 0.89). However, the multivariate Levene’s test indicated that the dispersion among the three types of figs was significantly heterogeneous (F_2,13_ = 5.15, *P* = 0.02). This variation was mainly due to the significant difference in dispersion between spring males and summer males (*P* = 0.01) and between spring males and females (*P* = 0.005). In contrast, the variation of scents emitted by female and summer male figs was homogeneous (*P* = 0.78). Finally, the total quantity of the four antennal-active VOCs emitted (see Table 1) was significantly different among the three types of figs (F_2,13_ = 9.81, *P* = 0.004). This effect was mainly due to the significant difference in the total quantity emitted by figs between spring males and summer males (*P* = 0.02) and between spring males and females (*P* = 0.005). In contrast, the variation of total scents emitted by female and summer male figs was not significantly different (*P* = 0.73).

### Which VOCs are responsible for pollinator attraction?

**Figure 5:**
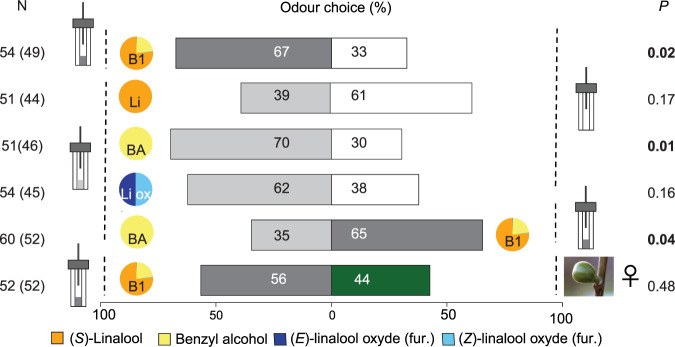
Attraction responses of *Blastophaga psenes* females towards different VOCs alone or in blends. Tests were conducted in Y-tube olfactometers in which females were allowed to choose between synthetic versions of the four VOCs (alone or in a blend) and control odour. In a second set of tests, female wasps could select between a blend of the four VOCs and benzyl alcohol alone, or, a blend of the four VOCs and odour of female receptive figs. For the four VOCs detected by the pollinator, proportions of each in each odour source are indicated in the pie chart and in more details in Table 1. Number of wasps tested (N), number of individuals that made a choice in parentheses, and *P*-values (exact binomial test) are indicated for each comparison.

In the bioassays with Y-tube olfactometers, females of *B. psenes* were significantly more attracted by the blend 1 (B1, the blend with VOC proportions are similar to the mean of the three types of receptive figs; Table 1) compared to the control (Fig. 5, binomial test, N = 49, *P* = 0.02), whereas wasps did not show any preference when given the choice between B1 and a female receptive fig (Fig. 5, binomial test, N = 50, *P* = 0.48). They were not preferentially attracted to either the racemic mixture of linalool (binomial test, N = 44, *P* = 0.17) or the mixture of (Z) and (E)-linalool oxide furanoid (binomial test, N = 45, *P* = 0.16) when compared to the control (Fig. 5). Whereas wasps were more attracted to benzyl alcohol alone compared to the control (Fig. 5, binomial test, N = 46, *P* = 0.01), they nonetheless preferred B1 compared to benzyl alcohol alone (Fig. 5, binomial test, N = 52, *P* = 0.04).

**Figure 6:**
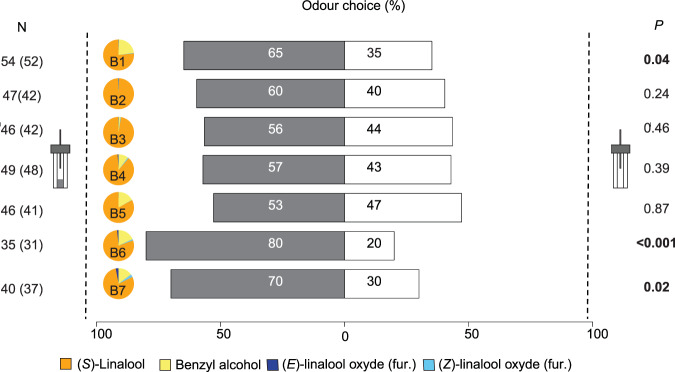
Attraction responses of *Blastophaga psenes* females towards blends with different proportions of the three VOCs. Tests were conducted in Y-tube olfactometers in which female wasps were allowed to choose between synthetic versions of the four VOCs in different proportions, in one branch, and control odour in the other. For the four VOCs detected by the pollinating females, proportions in each odour source are indicated in the pie chart and in Table 1. Number of wasps tested (N), number of individuals that made a choice (in parentheses), and *P*-values (exact binomial test) are indicated for each comparison.

When blends were tested against an empty container (control), wasps were significantly attracted to (Fig. 6, binomial test): B1 (N = 52, *P* = 0.04), B6 (N = 31, *P* < 0.001) and B7 (N = 40, *P* = 0.02). None of the other blends (*i.e*. VOC proportions differing substantially from those in receptive figs; Table 1) was more attractive than the control (Fig. 6, binomial test): B2 (N = 42, *P* = 0.24), B3 (N = 42, *P* = 0.46), B4 (N = 48, *P* = 0.39) and B5 (N = 41, *P* = 0.87).

## Discussion

This study provides new insights into the chemical ecological basis of host plant recognition in interactions between figs and their pollinating fig wasps, and in other highly specialized plant-pollinator interactions. We showed that, as hypothesized, *B. psenes*, the pollinator of the Mediterranean *Ficus* species, *F. carica*: 1) does not show any preference for the odour of either male or female receptive figs in summer when they are partly co-flowering; 2) detects only five of the 26 VOCs emitted by receptive figs; 3) is attracted by the blend of four of these VOCs in a specific set of proportions.

*Blastophaga psenes* uses to locate its obligatory host a blend of four VOCs commonly emitted by plants [benzyl alcohol, (*S*)-linalool, (*Z*)-linalool-oxide (furanoid), and (*E*)-linalool-oxide (furanoid)]. The results of our behavioural tests revealed that the presence of these four VOCs in the blend is crucial for wasp attraction and a decrease of the proportions of benzyl alcohol or of the linalool-oxides in the blend reduces its attractiveness to the pollinator. Therefore *B. psenes* needs a particular proportion of these four common VOCs to locate its host plant. Interestingly, similar mechanisms are well known in another kind of extremely specialized interactions, sexual recognition between insects. For instance, in several moth species, females emit species-specific pheromones that attract conspecific males over long distances while inhibiting the attraction of sympatric heterospecific males^32,33^. This mechanism therefore plays a significant role in interspecific reproductive isolation. Such specificity is achieved by a combination of VOCs, generally fatty-acid compounds, emitted by females in a unique blend with particular proportions^32^. Males are attracted by this signal and a small change in the proportion of these VOCs can have a critical impact on male behaviour^34,35^. In contrast, little is known about the coding of the chemical signal mediating most plant-insect interactions. Based on studies conducted on model systems (*e.g. Drosophila melanogaster*, *Apis mellifera* or *Manduca sexta*) it has been suggested that insects in general use particular proportions of multiple VOCs to find their resources in the chemical landscape^4,36,37^. However, this hypothesis has been rarely tested empirically, as most of the studies investigating the signal mediating plant-insect interactions compare insect responses to blends of different VOCs^38–40^ but rarely carry out bioassays with different proportions of these VOCs in the blend^41,42^. A major improvement in the study of signals mediating plant-insect interactions would come if attraction tests with different proportions of VOCs were conducted, as has been done to test conspecific recognition in moths^34,35^, in order to characterize the extent to which the proportions of the different constituents could be changed without affecting insect attraction. In addition, in contrast to what we have done in the current study, in almost all previous studies that have tested blends of VOCs, there is a lack of measurement of the diffusion rate of the different compounds and of the stability of their proportions in the resultant signal. VOCs have considerably different evaporation rates^43^ and as a consequence, in mixtures of different VOCs, their proportions diffused in the odour source should be very different from their proportions initially applied in the diffuser.

Our results indicate that a very small change in the proportions of the four VOCs could impede the attraction of females of *B. psenes*. Scent emitted by flowers has conventionally been viewed as a trait that is highly variable within one species, based on genetic drift, environmental constraints or selection mediated by pollinators or other agents^44,45^. However, in the case of extremely specialized plant-pollinator interactions, the fitness of both partners is strongly dependent on the maintenance of the interaction. Thus, it is expected that the chemical signal responsible for pollinator attraction should be under strong stabilizing selection^12^. To our knowledge, *B. psenes* is the sole pollinator of *F. carica* throughout its distribution. Whereas there should be strong selection to conserve the proportions of the VOCs emitted by *F. carica* receptive figs that contribute to attraction of *B. psenes*, we should observe more variation in the proportions of those VOCs that do not contribute to attraction, as has been previously reported for some orchid species^12^. Collections and analyses of the VOCs emitted by different populations of *F. carica* would be required in order to test this hypothesis.

*Blastophaga psenes* is not preferentially attracted by the VOCs emitted by summer male figs compared to those emitted by female figs. To our knowledge, this is the first study that demonstrates clearly that a species of pollinating fig wasp associated with dioecious figs does not show any preference for the VOCs emitted by male figs of its host compared to those emitted by conspecific deceptive female figs. Although our experiments do not allow affirming whether wasps can or cannot differentiate male from female figs, they reveal that *B. psenes* individuals do not prefer one to the other. These results are consistent with the hypothesis that intersexual chemical mimicry of odours of male and rewardless female figs prevents choice by pollinators when they are partly co-flowering during summer^3,26–28^.

A previous study analysing the overall scent of receptive figs of *F. carica* reported a significant difference in the relative proportions of all the VOCs emitted by spring male figs compared to summer figs, and more specifically an increase in the relative proportions of several sesquiterpenes in summer^29^. Nonetheless, that study did not investigate whether such a difference exists also for the specific VOCs used by the pollinator as signal. Our study reveals that the mean relative proportions of the four VOCs used by the pollinator (one shikimic compound and four monoterpenes) are not significantly different among the three types of figs. Contrary to the quantity of sesquiterpenes in the overall fig scent^29^, these four VOCs are emitted in much higher quantities in spring figs than in summer figs (16 times more than in female figs and 4 times more than in male figs). These inter-seasonal differences in the total quantity of VOCs, both those involved in the attraction of the pollinator and others, could be driven by abiotic or biotic factors, or by the two combined. Indeed, it is well established that environmental conditions, particularly temperature, can affect VOC emission by plants^46,47^. On the one hand, owing to the physicochemical properties of plant VOCs, warming increases the rates of total emissions of VOCs in plants^44,46^. However, this cannot explain the variation in our study, as temperatures in the Mediterranean region are higher in summer than in spring. On the other hand, in response to variable temperature conditions, floral physiology can modify the biosynthetic activity of terpene synthases to regulate the emission of each floral compound, or of multiple compounds simultaneously, depending on synthase specificity^48^. As a consequence, in addition to affecting the overall quantity of VOCs emitted by flowers, temperature can also change the proportions of the compounds that constitute floral scents. For instance, in a community of Mediterranean plants, a species-specific optimum temperature has been reported in the emission of VOCs by flowers, and temperature also affects the relative proportions of different compounds^49^. Another possible explanation for the observed inter-seasonal variation is that it results from pollinator-mediated selection acting differently on floral scent in spring and summer figs. Indeed, pollinator density is considerably lower in spring compared to summer^30,50^, leading to greater competition between individual trees for access to pollinators in spring than in summer. As a consequence, selection should favour male fig phenotypes that emit a larger amount of the VOCs to attract pollinators in spring, when pollinators are a more strongly limiting resource. In other plant-pollinator interactions, selection to increase the emission of VOCs detected by pollinators has been reported^51^. In summer, pollinator-mediated selection in relation to VOCs emission by male figs is expected to be different, because (i) pollinator density is higher than in spring and (ii) summer figs are partly co-flowering with rewardless female figs. Each sex may be under vicarious selection, *i.e*. selection acts on each sex to resemble the other, in order to prevent the breakdown of the interaction. Because intersexual mimicry benefits pollination and assures seed production (but see^28,29^), we do not expect that selection will favour an increase in the emission of VOCs by male figs during summer.

Coding of the signal responsible for plant-pollinator encounters has been elucidated in only a limited number of other obligate nursery pollination mutualisms^6,16,17^. The present study reveals that females of *B. psenes* do not use a rare compound to localize the host plant but a specific blend of common VOCs in very particular set of proportions. The wasp family Agaonidae is estimated to include more than 1000 species^52^. Each species of Agaonidae is associated with one or very few *Ficus* spp. and is specifically attracted by the VOCs emitted by the receptive figs of its host(s)^3,6,18,20,21,23^. Chemical analyses conducted so far on more than 30 *Ficus* spp. have revealed that species of this genus emit species-specific mixtures of VOCs that are usually commonly emitted by flowers^3,18,21,23–25,28^. For instance, more than 50% of *Ficus* species emit the monoterpenes (E)-β-ocimene and linalool or the sequiterpenes α-copaene, α-humulene or germacrene D, which are very commonly emitted by plants^3,18,21,23–25,28,31^. Therefore, we can expect that, similarly to *B. psenes*, the majority of species of Agaonidae use a particular combination of common VOCs in unique proportions, and not uncommon compounds, to localize their host plant. Studies similar to the present one, combining chemical and electrophysiological analysis with behavioural tests, should be conducted in the future in order to establish if our findings can be generalized, not only to interactions between other figs and their pollinating fig wasps, but also to other nursery pollination mutualisms.

## Materials and Methods

### Study system

This study was carried out in the region of Montpellier, southern France, with insects from natural populations collected in fig trees present at the CEFE (“Centre d’Ecologie Fonctionnelle et Evolutive”) experimental garden (43°38′19″N, 3°51′49″E) in Montpellier, France, and from natural populations less than 40 km distant from Montpellier.

Our model system is the mutualistic interaction between the agaonid *Blastophaga psenes* L. (Hymenoptera, Chalcidoidea, Agaonidae) and its exclusive host, the Mediterranean *Ficus* species, *i.e. Ficus carica* (subgenus *Ficus*, section *Ficus*, subsection *Ficus*). This pollinating fig wasp species has two generations per year coinciding with the flowering of male trees first in April (“spring male figs”) and then in July (“summer male figs”) (Fig. 1). In contrast, female trees flower only once a year, in July, and thus partially synchronously with summer male figs (Fig. 1). Detailed life cycles of both *Blastophaga psenes* and *Ficus carica* are presented in Fig. 1 and in^50,53^.

### Preparation of odour sources

As for all the analysis and behavioural tests, odour preparations were conducted at the “Platform for Chemical Analyses in Ecology” (PACE, Montpellier) technical facilities of the LabEx CeMEB (“Centre Méditerranéen pour l’Environnement et la Biodiversité”, Montpellier, France). For the GC-EAD recordings, VOCs of receptive summer male figs and female figs were collected using classical adsorption-desorption headspace technique^30,54,55^. Three groups of 20 to 30 receptive figs were collected haphazardly from both male and female trees and directly enclosed in polyethylene terephthalate bags. For each bag, traps containing 30 mg of Alltech Super Q adsorbent (ARS Inc., Gainesville, FL, USA) were placed at the end of tube from which air was drawn in. Airflow was maintained through the bags by two pumps (KNF, Neuberger, Freiburg, Germany). Air pushed into the bag by a polytetrafluoroethylene (PTFE) tube was filtered using activated charcoal. The entrance and exit flow rates were regulated by flowmeters at 300 and 200 ml·min^−1^, respectively, to create a positive pressure inside the bag and thereby prevent contamination from the environment. The collection duration was 4 h. Each trap was eluted with 150 μl of hexane (Sigma Aldrich, Munich, Germany, purity >99%) and the three extracted samples were pooled together and stored at −20 °C. In addition to these odour samples, synthetic compounds were used during the electrophysiological recordings to validate the antennal responses.

For the behavioural tests, summer male figs, female figs, and synthetic compounds, singly or in blends, were used. Fresh male and female figs were collected in the field and used within 2 h after collection for the behavioural tests. For these bioassays, a single fig was used for each test. Furthermore, four synthetic compounds were chosen based on their activity on insect antennae (see below) and their constant occurrence in the volatile profile of receptive figs of *F. carica*^29,30^. With these compounds, seven blends were prepared with different compound proportions (Table 1). The composition of the blend 1 (B1) was based on the average proportion and quantities of the four synthetic compounds emitted by the three types of figs (Table 1), with the constraint that some VOCs were not available commercially as pure compound. In order to investigate the importance for pollinator attraction of each compound in the blend, in the following six other blends we reduced or increased the proportions of the different VOCs. To deliver the odours in the experimental set-up (Supplementary, Fig. S1), pure synthetic compounds were added into a glass insert of 400 μl, which was placed into a 1.5 ml vial sealed with a polytetrafluoroethylene/rubber septum (Chromoptic, Courtaboeuf, France). A micro-capillary tube made of fused silica (Agilent technologies, Redmond, USA), 40 mm long and 0.53 mm internal diameter (ID), was inserted through the septum. The diameter and length of the capillary tube were calibrated to release a controlled amount of VOCs corresponding to the mean release rates of one receptive fig. For that, vials were positioned inside glass containers of 500 ml and a diameter of 100 mm that were connected to a continuous airflow of 200 ml.min^−1^ and maintained inside an oven at 25 °C. Vials were weighed regularly during 2 months using a microbalance (MC5, Sartorius, Goettingen, Germany) to determine the diffusion rate (ng.min^−1^). Preliminary tests revealed that diffusion of the VOCs using these dispensers is only stable after 10 days (Proffit *et al*., *unpublished data*).

### Electrophysiology on *B. psenes*

GC-EAD recordings were conducted on a gas chromatograph-flame ionization detector (GC-FID, CP-3800, Varian, Palo Alto, USA) equipped with an optima 5-MS capillary column (30 m, 0.25 mm ID, 0.25 μm film thickness, Machery-Nagel, Düren, Germany) coupled to an electroantennography detector setup (EAD, Syntech IDAC-2, Kirchzarten, Germany). Four μl of either receptive fig odour or synthetic mix solution were injected into the GC-FID. The injector was heated to 250 °C, with a 1:4 split ratio to inject the compounds into the column. Oven temperature was held at 50 °C for 1 minute, increased from 50 °C to 100 °C at a rate of 9 °C.min^−1^ then from 100 to 140 °C at a rate of 8.1 °C.min^−1^, then from 140 °C to 190 °C at a rate of 7.2 °C min^−1^, then from 190 °C to 210 °C at a rate of 20 °C.min^−1^ and finally the temperature was held at 210 °C during 50 seconds. The carrier gas used was helium at 1 ml·min^−1^. The effluent was split equally into two deactivated fused silica capillary columns (100 cm × 0.25 mm), one leading to the FID (270 °C) and one into a heated EAD port (200 °C) (transfer line, Syntech, Kirchzarten, Germany). For the EAD, wasp heads were cut at their base. Head base and the tip of one antenna were mounted between two glass capillary tubes filled with insect Ringer solution (6.0 g·l^−1^ NaCl, 0.4 g·l^−1^, KCl, 0.27 g·l^−1^, CaCl_2_ and 3.20 g·l^−1^ of sodium lactate) and connected to silver wires. Electrophysiological measurements were conducted separately with the antennae of seven adult female wasps for each odour source tested. A compound was considered to be EAD-active when it elicited an unequivocal depolarization response in four antennae out of seven. In addition, the activity of VOCs on pollinator antennae was confirmed using synthetic standards.

### Volatile collections and chemical analysis

Headspace collections of VOCs of spring male (N = 6), summer male and female receptive figs (N = 4 for each), and of all the dispensers used for the behavioural test were conducted. For these collections, either receptive figs or an odour dispenser were placed into a glass container of 500 ml for 30 minutes before collection. Air pushed into the glass container was filtered using activated charcoal at a flow rate of 200 ml·min^1^. The same amount of air was drawn out of the container through an adsorbent trap compatible with a thermal desorption system, consisting of an external glass tube (length: 60 mm and 6 mm O.D., Gerstell, Mulheim, Germany) filled with 80 mg of Tenax-TA and 40 mg of Carbotrap (60–80 and 20–40 mesh respectively, Sigma-Aldrich, Munich, Germany). Odour collections lasted 10 min for the synthetic compounds and 30 min for the figs. All adsorbent traps were sealed with lids on both side openings and stored at −20 °C until further use.

Chemical analyses were conducted using a method similar to that of Souto-Vilarós *et al*.^18^. We used a gas chromatograph (GC, Trace 1310, Thermo Scientific, Milan, Italy) coupled to a mass spectrometer (ISQ QD Single Quadrupole, Thermo Scientific, Milan, Italy). The column used was an Optima 5-MS capillary column (30 m, 0.25 mm ID, 0.25 μm film thickness, Machery-Nagel, Düren, Germany). Absorbent traps were handled with a Multi Purpose Sampler (Gerstell, Mülheim, Germany) and desorbed with a double stage desorption system, composed of a Thermal Desorption Unit (TDU) and a Cold Injection System (CIS) (Gerstell, Mülheim, Germany). First, the injector was splitless with a temperature of 250 °C on the CIS trap cooled at −80 °C by liquid nitrogen. Then, the CIS trap was heated to 250 °C with a 1:4 split ratio to inject the compounds in the column. We used helium at 1 ml·min^−1^ as a carrier gas. Oven temperature was held at 40 °C for 3 minutes, increased from 40 °C to 220 °C at a rate of 5 °C·min^−1^ and from 220 to 250 °C at 10 °C·min^−1^, and finally held for 2 minutes at 250 °C. The temperature of the transfer line and the ion source of the mass spectrometer was 250 °C and 200 °C respectively. The acquisition was at 70 eV ionisation energy, from 38 m/z to 350 m/z. We used Xcalibur TM 266 software (Thermo Scientifc TM, Milan, Italy) for data processing. Retention times of a series of *n*-alkanes (Alkanes standard solution, 04070, Sigma Aldrich, Munich, Germany) were used to convert retention times into a retention index. Compound identification was based on computer matching of mass spectra and retention indices with reference compounds. In addition, to quantify precisely the emission rate of the VOCs detected by the pollinator in each sample, known amounts of these different reference compounds (100, 50, 20 and 2 ng) were injected into an adsorbent trap and analysed in the GC-MS system using the same method. Their mean peak areas were used for calibration. In addition, the stereochemistry of linalool was determined using the same analytic method as indicated above in the same GC-MS equipped with a β-cyclodextrin chiral capillary column (Cyclosil-B, 30 m–0.25 mm i.d., 0.25 μm film thickness, Agilent J&W columns, USA).

### Behavioural experiments

We tested whether females of *B. psenes* were attracted to VOCs emitted by receptive figs of *F. carica* and by several synthetic compounds, using a glass Y-tube olfactometer in which only chemical cues were presented to wasps (Supplementary, Fig. S1)^6,18,23^. Bioassays were conducted from 2016 to 2019 under laboratory conditions at the temperature of 25.75 ± 0.10 °C and relative humidity of 51.52 ± 1.60%. Y-tubes were in glass (40 mm in diameter), each lateral arm was 200 mm long and the central arm was 150 mm long. Odour sources were placed in glass containers of 500 ml and connected to each lateral arm of the Y-tube. Air was purified with activated charcoal, humidified with distilled water and blown into the glass containers (200 ml·min^−1^ per arm). Y-tubes and glass containers were changed and cleaned with acetone after each trial in order to remove any chemical traces left by the insects. To avoid a directional bias, the positions of odour sources were inverted between the two arms in each successive trial. Treatments were alternated to be able to compare wasp choice frequencies between treatments. For the bioassays, between 42 and 60 fig wasps were tested per treatment. Air was blown for 1 min prior to insect introduction into the olfactometer. During ten minutes, the choice made by each individual was recorded. We considered that wasps did not choose when they stayed motionless in the departure section and/or the central arm before the bifurcation of the olfactometer after these ten min. These wasps were not taken into account in the statistical analyses.

Newly emerging adult female wasps were collected from mature figs taken haphazardly from different individual male trees. Because of their very short lifespan outside the fig (less than 24 h), individuals of *B. psenes* were tested shortly after their exit. Each day a maximum of 25 individuals were tested per treatment. All tested wasps were naïve to the odour sources presented in our bioassays.

### Data analysis

All the data analyses were performed in R (v. 3.5.3; R Development Core Team; http://www.R-project.org) using multivariate analysis incorporated in the Vegan package^56^. As variation in the relative proportions of all the VOCs emitted by receptive figs of *F. carica* was compared in a previous study^29^, we only focused on comparison among the three types of receptive figs of relative proportions of the four VOCs used by the pollinator. Data were standardized before the analyses and a data matrix of pairwise Bray-Curtis dissimilarity indices between samples was built. Non-metric multi-dimensional scaling (NMDS) was used to visualize similarities among the samples by finding the best two-dimensional representation of the distance matrix. A Permutational Multivariate Analysis of Variance (PERMANOVA) based on 999 permutations was used to test the null hypothesis of no centroid (*i.e*. mean) difference in on the relative proportions of the four VOCs among the three different types of figs, as well as in pairwise comparisons. The difference of dispersion (*i.e*. variance) in the relative proportions of these VOCs among the three different types of figs, and also in pairwise comparisons, was tested using a multivariate analogue of Levene′s test for homogeneity of variance. Finally, we performed an ANOVA to compare the total amounts of VOCs emitted by receptive figs of the three types. For pairwise comparisons, *P*-values were adjusted for multiple comparisons using the FDR method^57,52^.

For all dual-choice bioassays in the Y-tube olfactometer, two-tailed exact binomial tests were used to test the null hypothesis that the same number of wasps was attracted to both odour sources.

## Supplementary information

Supplementary Information.

Supplementary Information 2.
