# Author Correction: Chemical signal is in the blend: bases of plant-pollinator encounter in a highly specialized interaction

**DOI:** 10.1038/s41598-020-75642-0

**Published:** 2020-11-09

**Authors:** Magali Proffit, Benoit Lapeyre, Bruno Buatois, Xiaoxia Deng, Pierre Arnal, Flora Gouzerh, David Carrasco, Martine Hossaert-McKey

**Affiliations:** 1 EPHE, IRD, CEFE, Univ Montpellier, CNRS, Univ Paul Valéry Montpellier 3, Montpellier, France; 2 MIVEGEC, Univ Montpellier, IRD, CNRS, Montpellier, France

Correction to:* Scientific Reports* 10.1038/s41598-020-66655-w, published online 22 June 2020

The original version of this Article contained an error in the spelling of the author Xiaoxia Deng, which was incorrectly given as Xia-Xio Deng.

As a result, in the Acknowledgements,

“Xia-Xio Deng’s PhD grant was supported by China Scholarship Council [No. (2017)3109].”

now reads:

“Xiaoxia Deng’s PhD grant was supported by China Scholarship Council [No. (2017)3109].”

In the Author Contributions,

“M.P., B.L., X.X.D., P.A. and F.G. collected the data.”

now reads:

“M.P., B.L., X.D., P.A. and F.G. collected the data.”

These errors have now been corrected in the PDF and HTML versions of the Article.

