# Supplementary material for: Chemical signal is in the blend: bases of plant-pollinator encounter in a highly specialized interaction
Source: Sci Rep. 2020 Jun 22;10:10071. doi: 10.1038/s41598-020-66655-w (PMC7308319; doi:10.1038/s41598-020-66655-w)
Supplement: Supplementary file 2 — Supplementary Information 2. [file 41598_2020_66655_MOESM2_ESM.docx]

**Supplementary file**

**Chemical signal is in the blend: bases of plant-pollinator encounter in a highly specialized interaction**

Magali Proffit, Benoit Lapeyre, Bruno Buatois, Xiaoxia Deng_,_ Pierre Arnal, Flora Gouzerh, David Carrasco, Martine Hossaert-McKey

**Figure legend**

**Figure sp1: Schematic of the system used to conduct behavioral tests with Y-tube olfactometer.** Details on the equipment and protocol used to test wasp attraction with this set-up are presented in Materials and methods/Behavioral experiments. “VOCs” stands for volatile organic compounds and “ctrl” for control. This figure was prepared with the help of Jennifer McKey.
